# Current status and developments of German curriculum-based residency training programmes in radiation oncology

**DOI:** 10.1186/s13014-021-01785-7

**Published:** 2021-03-20

**Authors:** Marcel Büttner, Nils Cordes, Tobias Gauer, Daniel Habermehl, Gunther Klautke, Oliver Micke, Matthias Mäurer, Jan Sokoll, Esther Gera Cornelia Troost, Hans Christiansen, Maximilian Niyazi

**Affiliations:** 1 Department of Radiation Oncology, University Hospital, LMU Munich, Marchioninistraße 15, 81377 Munich, Germany; 2 German Cancer Consortium (DKTK), Partner Site Munich, Munich, Germany; 3 Department of Radiotherapy and Radiation Oncology, Faculty of Medicine and University Hospital Carl Gustav Carus, Technische Universität Dresden, Dresden, Germany; 4 OncoRay-National Center for Radiation Research in Oncology, Faculty of Medicine and University Hospital Carl Gustav Carus, Technische Universität Dresden, Helmholtz-Zentrum Dresden-Rossendorf, Dresden, Germany; 5 National Center for Tumor Diseases (NCT), Partner Site Dresden, Dresden, Germany; 6 German Cancer Research Center (DKFZ), Heidelberg, Germany; 7 Faculty of Medicine and University Hospital Carl Gustav Carus, Technische Universität Dresden, Dresden, Germany; 8 Helmholtz Association/Helmholtz-Zentrum Dresden - Rossendorf (HZDR), Dresden, Germany; 9 Helmholtz-Zentrum Dresden-Rossendorf, Institute of Radiooncology - OncoRay, Dresden, Germany; 10 Department of Radiotherapy and Radio-Oncology, University Medical Center Hamburg-Eppendorf, Hamburg, Germany; 11 Radprax MVZ, Leimbacher Str. 51a, 42281 Wuppertal, Germany; 12 Clinic for Radiation Oncology, Chemnitz Medical Center, Chemnitz, Germany; 13 Department of Radiotherapy and Radiation Oncology, Franziskus Hospital Bielefeld, Kiskerstrasse 26, 33615 Bielefeld, Germany; 14 Department of Radiation Oncology, University Medical Center Jena, Jena, Germany; 15 PRO RadioOncology GmbH, Poststraße 10-12, 27404 Zeven, Germany; 16 Department of Radiation Oncology, Hannover Medical School, 30625 Hannover, Germany

**Keywords:** Radiotherapy, Radiation oncology, Curriculum, Training, Evaluation, DEGRO

## Abstract

**Purpose:**

The current status of German residency training in the field of radiation oncology is provided and compared to programmes in other countries. In particular, we present the DEGRO-Academy within the international context.

**Methods:**

Certified courses from 2018 and 2019 were systematically assigned to the DEGRO-Curriculum, retrospectively for 2018 and prospectively for 2019. In addition, questionnaires of course evaluations were provided, answered by course participants and collected centrally.

**Results:**

Our data reveal a clear increase in curriculum coverage by certified courses from 57.6% in 2018 to 77.5% in 2019. The analyses enable potential improvements in German curriculum-based education. Specific topics of the DEGRO-Curriculum are still underrepresented, while others decreased in representation between 2018 and 2019. It was found that several topics in the DEGRO-Curriculum require more attention because of a low DEGRO-curriculum coverage. Evaluation results of certified courses improved significantly with a median grade of 1.62 in 2018 to 1.47 in 2019 (*p* = 0.0319).

**Conclusion:**

The increase of curriculum coverage and the simultaneous improvement of course evaluations are promising with respect to educational standards in Germany. Additionally, the early integration of radiation oncology into medical education is a prerequisite for resident training because of rising demands on quality control and increasing patient numbers. This intensified focus is a requirement for continued high standards and quality of curriculum-based education in radiation oncology both in Germany and other countries.

**Supplementary Information:**

The online version contains supplementary material available at 10.1186/s13014-021-01785-7.

## Introduction

The Academy of the German Society for Radiation Oncology (DEGRO-Academy) was founded in 2004. The principle tenant of the DEGRO-Academy is Continuous Medical Education (CME), implemented through the standardisation of medical specialist training in the field of radiation oncology [1].

The aim of the DEGRO-Academy is the achievement of a homogeneous specialist training across Germany, thus ensuring adequate patient care on a national scale. The need for an improvement of both organisation in specialist training and quality, as well as comparability on a national scale was shown by Semrau et al. [2]. In the subject area of ionizing radiation their training courses have already benefitted from such standardisation [3].

The DEGRO-Academy certifies educational courses that fulfil the high organisational and qualitative requirements of the society. Additionally, the DEGRO-Academy introduced the curriculum required to become a resident in radiation oncology (DEGRO-Curriculum). This curriculum was designed and approved by a board of German radiation oncology experts [4].

Prior to the introduction of a national curriculum by the DEGRO-Academy, residents in radiation oncology were only subject to the specialty training regulations of the respective state chamber of physicians ("Landesärztekammer") (i.e. Baden-Württemberg [5] and Bavaria [6]) which are now supplemented by the DEGRO-Curriculum.

A national interdisciplinary survey by the German Medical Association ("Bundesärztekammer") proved that radiation oncologists are satisfied with their respective training programmes in radiation oncology between 2009 and 2011 [7, 8]. However, in a recent study conducted by the "young DEGRO” (yDEGRO) in 2018 64% reported that their residency would benefit from a standardised curriculum (n = 96, median age of participants 31 years) [9]. The working group yDEGRO, which is comprised of young members of the DEGRO, is not only involved in education, but also independent in research, for instance in the field of lung cancer [10].

A second study reported that 47.2% of supervising physicians in the training of radiation oncology residents reported large difficulties in finding candidates [11].

The DEGRO-Academy revised its curriculum while building on the results of the study by the yDEGRO. The curriculum complements the current fourth Edition of the "Core Curriculum for Radiation Oncology/Radiotherapy" by the European Society for Radiotherapy and Oncology (ESTRO) [9, 12, 13]. The aim of the DEGRO-Curriculum is to give residents a comprehensive standardised education without replacing the respective, pre-existing state chamber of physicians guidelines (i.e. Baden-Württemberg [5] and Bavaria [6]). Furthermore, in a previous study conducted by Röper et al. [14], certified refresher courses were analysed and a large interest of the participants in these certified courses was observed. In order to increase quality control, a standardised and comprehensive evaluation of all certified courses is required, thus ensuring a high educational benchmark.

The aim of this report is to determine the quantitative and qualitative development of certified courses after an evaluation of two years from 2018 to 2019, both internally and via an international comparison.

## Methods

### Curriculum

The data was collected on the basis of the latest curriculum, Version 2018 [4] (see Additional file 1: Curriculum of the DEGRO-Academy). In 2018, the assignment of course to curriculum was performed centrally by the DEGRO-Academy. In 2019 the assignment was performed by either the course director or the DEGRO-Academy. The curriculum data were analysed separately for each year.

### Evaluation

**Fig. 1:**
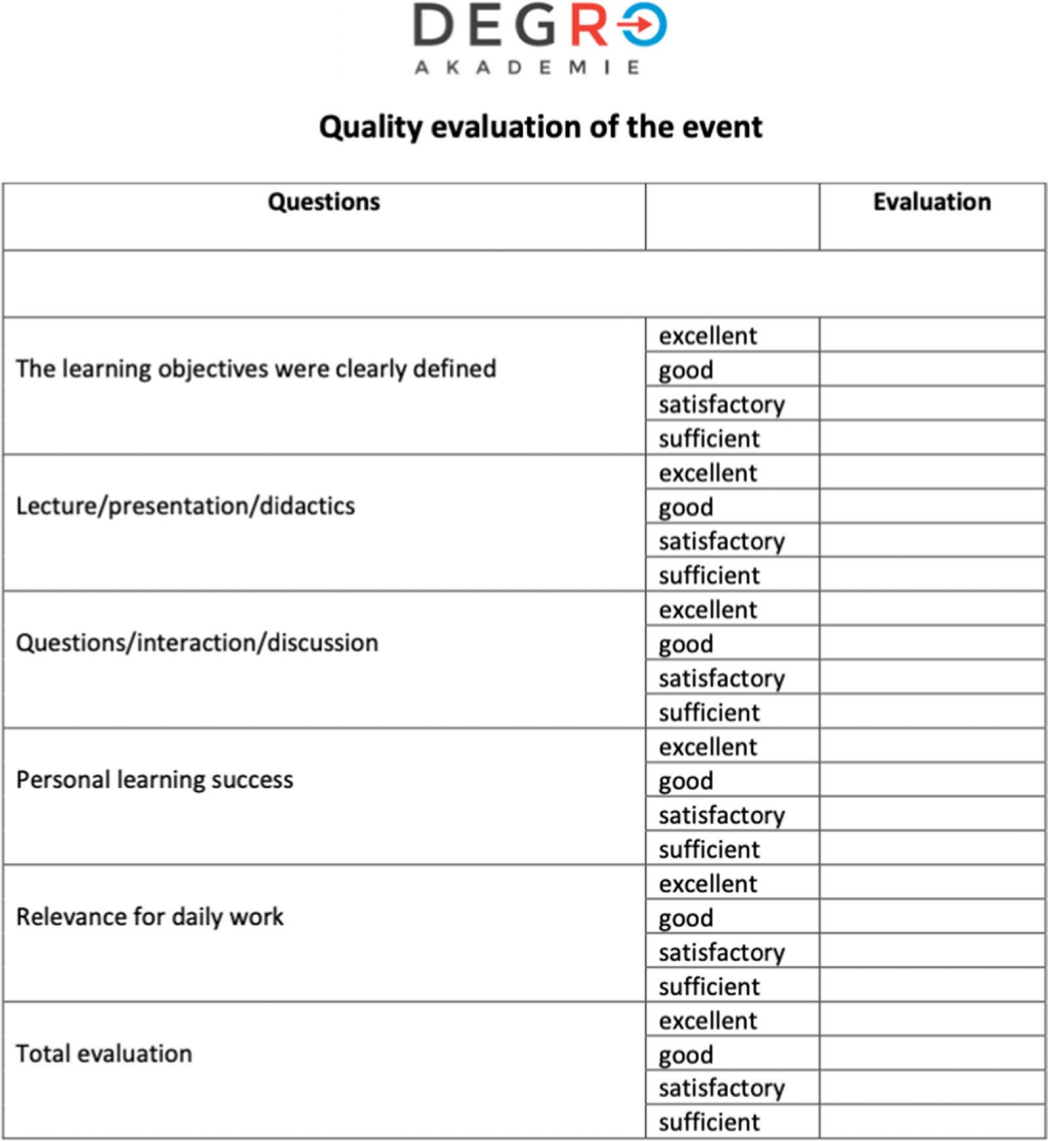
Standardised questionnaire of the DEGRO-academy

The survey was an individual evaluation by the organizers, conducted on site or by mail invitation between March 2018 and December 2019. Questionnaires were provided by the DEGRO-Academy and ensuring their completion was mandatory for all event organisers. After finalisation of the questionnaires by the participants these were collected centrally. The questionnaire consisted of six items assessing didactic quality (teaching quality), personal growth of knowledge (knowledge improvement) relevance in the clinical setting (implication in everyday’s practice), clarity of defined learning objectives (clarity of subject material) and a total evaluation (overall score) of the course (Fig. 1). Additional questions pertained to the relevance of the course to current research and the professional backgrounds of the participants. Answers were rated on a nominal scale from 1 (best) to 4 (worst).

### Statistics

The raw data of assignment of course to curriculum and evaluation sheets were collected and stored in the online data base ForMES (Version 2.210, ForMES Service GmbH, Fehmarn, Schleswig-Holstein, Germany). For further analysis we used GraphPad Prism (Version 8, GraphPad Software, San Diego, CA, USA) and Microsoft Excel (Mac Version 16.40, Microsoft, Redmond, WA, USA). Unless otherwise indicated, percentages were rounded off to the nearest integer.

## Results

### Curriculum

**Fig. 2:**
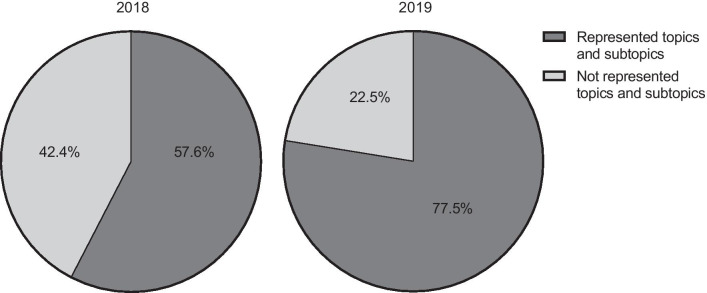
Percentages of represented DEGRO-Curriculum courses in 2018 and 2019, which were certified by the DEGRO-Academy (n = 50 for 2018; n = 54 for 2019)

In 2018, certified courses achieved an overall coverage of 57.6% of the curriculum topics and subtopics. The course to curriculum assignment was based on a total of 50 courses. By comparison, the coverage rate in 2019 was 77.5% for a total of 54 assigned courses (Fig. 2). Thus, a higher percentage of DEGRO-Curriculum topics and subtopics in DEGRO-Academy certified courses were offered to residents in 2019 than in the previous year.

A further evaluation was initiated to find out which topics in the DEGRO-Curriculum were particularly over- or underrepresented (Table 1). Aside from an increase in the total representation from 2018 to 2019, all the subtopics of the topics "01. Radiobiology", "02. Physics and radiation protection" and "06. Radiotherapy of benign diseases" were represented in certified courses.

**Table 1:** Curriculum topics of the DEGRO-Academy with number of respective subtopics (with a maximum of 2 subclassifications) and number of represented subtopics from the DEGRO-Curriculum in certified courses in 2018 and 2019 (n = 50 for 2018; n = 54 for 2019)

Topics from the DEGRO-curriculum	Number of subtopics	Number of represented subtopics in certified courses and percentage of respective topic			
		2018 Number	%	2019 Number	%
01. Radiobiology	14	13	92.9	14	100.0
02. Physics and radiation protection	31	21	67.7	31	100.0
03. Radiation techniques	23	17	73.9	19	82.6
04. Classification of acute and late reactions, supportive therapy	22	11	50.0	17	77.3
05. Palliative radiation oncology	30	11	36.7	23	76.7
06. Radiotherapy of benign diseases	19	14	73.7	19	100.0
07. Malignant oncological organ-related tumor entities, incl. radiochemotherapy and targeted drugs	74	35	47.3	56	75.7
08. Imaging in radiation oncology	22	10	45.5	17	77.3
09. BVDST-relevant subjects, billing/DRG	30	24	80.0	14	46.7
10. Other	11	3	27.3	4	36.4
Total	276	159	57.6	214	77.5

Furthermore, there was a strong increase in the number of covered palliative medicine subtopics (36.7% in 2018 to 76.7% in 2019). Particularly, certified courses in 2019 included more subtopics of "03. Radiation techniques", "04. Classification of acute and late reactions, supportive therapy", "07. Malignant oncological organ-related tumor entities, incl. radiochemotherapy and targeted drugs", and "08. Imaging in radiation oncology" than in 2018. By comparison, the topic "10. Other" which encompasses the subtopics "10.2. Prevention" and "10.3. Epidemiology, statistics and study planning" [4], indicated no significant change in representation between 2018 (27.3%) and 2019 (36.4%). The coverage of the topic "09. BVDST-relevant subjects, billing/DRG" decreased from 80.0% in 2018 to 46.7% in 2019.

### Evaluations

In 2018 a total of 50 courses took place and 8 were evaluated with a total of 213 participants. 21 out of these 50 courses were refresher courses hosted by the DEGRO-Academy at the annual meeting of the German Society for Radiation Oncology. In 2019, a total of 54 courses including 23 refresher courses took place, 52 of them were evaluated and one was excluded from this study, due to unstandardized items in the questionnaire. The total number of participants was 1,565. The exclusion was made on the basis of deviations in the questionnaire and a resulting lack of comparability to the remaining 51 courses. The score of each course was acquired by averaging the item "Total evaluation” from the participants of the respective course.

**Fig. 3:**
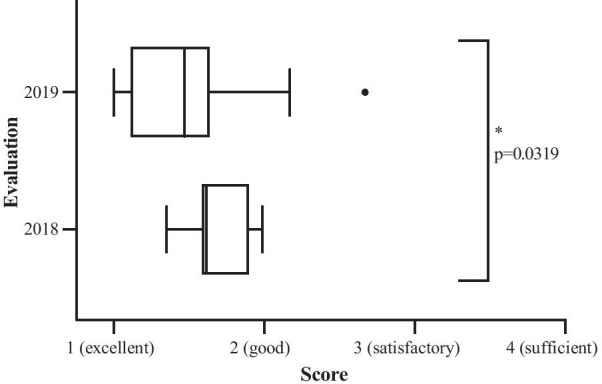
Comparison of the scoring between 2018 and 2019 (2018: 8 evaluated courses with 213 participants; 2019: 51 evaluated courses with 1565 participants; median score 2018: 1.62, 2019: 1.47; P value is significantly different with *p* = 0.0319, Mann–Whitney U test)

In 2018, the median score evaluated by 213 provided questionnaires from 8 courses was 1.62. In 2019 the evaluations of 1,565 questionnaires obtained a median scoring of 1.47 (Fig. 3). In both 2018 and 2019 none of the courses was scored with "sufficient". In summary, the scoring of the courses significantly improved between 2018 and 2019.

## Discussion

As the global demands for improved patient care and medical infrastructure in the field of radiation oncology increase, the quality and intensity of its specialists education must adapt to increasing requirements [15, 16]. At the same time the adaptation of educational courses to increasing knowledge is needed but presents a challenge [17–20]. Three out of ten topics ("01. Radiobiology", "02. Physics and radiation protection" and "06. Radiotherapy of benign diseases") listed in the DEGRO-Curriculum are already completely covered by certified courses, amongst them the topic "02. Physics and radiation protection". In comparison to a different online survey addressed to specialists in training by Dietzel et al. [9], the item "Evaluation of knowledge levels regarding basic topics" rated "02. Physics and radiation protection" to be the worst scored, with more than 60% of the participants rating it with "mediocre” or "bad”. The revision of course content and availability indicate a noticeable improvement in the area of residency training programmes and promises a better education for specialists. Furthermore, all subtopics of "01. Radiobiology" were represented in certified courses in 2019. A study in the US conducted by the American Society for Radiation Oncology (ASTRO) demonstrated in 2019 that, because of the importance of this area and the need for new graduate programmes focusing on radiobiology relating to radiation oncology, improved and advanced courses have to be developed [21].

Education in the field of palliative care is regarded as an important part of residency training programmes of radiation oncologists, since palliative medicine has a large impact on the daily working routine of physicians, as confirmed by Fels et al. [22] in 2019 analysing 205 eligible questionnaires. This was reflected by a substantial increase of certified courses covering this field between 2018 and 2019.

In addition, the coverage of "03. Radiation techniques" increased from 2018 to 2019. This topic encompasses modern techniques, such as stereotactic procedures and volumetric modulated radiotherapy (IMRT/VMAT), which have significantly increased [23–25]. A further component of this curriculum-topic is interventional radiotherapy, which has also become more accessible due to technical advancements, with an increasing number of brachytherapy treatments being recorded since 2004 [26, 27]. The need for an improvement of education in practical brachytherapy has also been reported in Italy, as shown in a recent study by Tagliaferri et al. [28] in 2019.

Other topics from the DEGRO-Curriculum such as "09. BVDST-relevant subjects, billing/DRG" and subtopics of "10. Other" require more certified courses in order to assure a broad education. Components of clinical research such as the subtopics "10.3. Epidemiology, statistics and study planning" and "10.5. Basics of evidence-based medicine" constitute part of this underrepresented area. An online survey by Krug et al. [29] of the yDEGRO showed that 70% of participants (260 survey respondents, 69% of them were medical doctors and the total median age was 33 years) had a particular interest in clinical versus preclinical/experimental (33%) and physical/technical (36%) research.

A European study by the ESTRO in 2020 concludes an urgent need for more support of innovative research by the national societies (58 national societies from 31 countries participated in this study) [30]. The need for more support was also documented by previous results from the Royal College of Radiology in 2012, where only 62% of the participants expressed their satisfaction with the degree of research education they gained during specialist training [31]. In Italy, this dissatisfaction was even more apparent after an evaluation with 197 participants (young radiation oncologists under the age of 40) conducted by Franco et al. in 2013. The authors report that most participants with high interest in clinical research (excellent/good: 70.6%) were unable to gain experience in this area (moderate/poor: 68%) [32]. A study by the Association of Residents in Radiation Oncology (ARRO) involving 135 radiation oncologists revealed that more than 50% of post-graduate year-5 radiation oncology residents had less than 6 months of research experience during their specialist training [33]. This international comparison underlines an urgent need to renew and improve several courses, particularly in of the subtopics "10.3. Epidemiology and statistics, study planning" and "10.5. Basics of evidence-based medicine" of "10. Other" (Table 1).

It may be deduced from the improvement of evaluation results from 2018 to 2019 that course participants do recognise and appreciate the course material and a higher quality of specialist training. Internationally, the importance of these certified courses and the associated CME is also recognised [30].

Another international comparison discussed large difficulties in the implementation of the ESTRO Core Curriculum on a nationwide scale. This year, a European study by Giuliani et al. [34] included 26 national societies from 26 different countries, reported complaints that governmental support is lacking in the implementation of the ESTRO Core Curriculum. Additionally, 44% of the participants were missing qualified staff members with professional teaching skills on the ESTRO Core Curriculum. In Germany, a similar observation was made in a study by Semrau et al. [2]. The authors came to the overall conclusion that out of 96 participants (members of the DEGRO and affiliated professionals), 60% suggested that there should be a stricter adherence to the training guidelines as laid out by the DEGRO-Curriculum, and 55% of the participants answered that more involvement by the DEGRO would prove beneficial.

Currently, radiation oncology plays an integral, but underrepresented role in the clinical education of medical students in Germany [35]. A study in the US suggests a further approach with a standardised curriculum for 4-week clerkships by medical students in the field of radiation oncology [36]. Another study by the ESTRO reported that the most important item in the education of radiation oncology is practical training (77% of participants) [37].

These findings indicate, that high quality education in the field of radiation oncology is extremely important to implement at an earlier stage of training in order to keep German radiation oncologists internationally competitive. The optimization of patient treatment requires the continued adjustment of the German healthcare system and the education of its specialists to increasingly high standards.

## Supplementary Information

**Additional file 1:** Curriculum of the DEGRO-Academy, Version 2018.

## Data Availability

Not applicable.

## Abbreviations

DEGRO-Academy: Academy of the German Society for Radiation Oncology
CME: Continuous Medical Education
DEGRO-Curriculum: The curriculum introduced by the Academy of the German Society for Radiation Oncology
yDEGRO: Young DEGRO
ESTRO: European Society for Radiotherapy and Oncology
ASTRO: American Society for Radiation Oncology
IMRT: Intensity-modulated radiotherapy
VMAT: Volumetric modulated radiotherapy
ARRO: Association of Residents in Radiation Oncology
