# Supplementary material for: Current status and developments of German curriculum-based residency training programmes in radiation oncology
Source: Radiat Oncol. 2021 Mar 20;16:55. doi: 10.1186/s13014-021-01785-7 (PMC7981823; doi:10.1186/s13014-021-01785-7)
Supplement: Supplementary file 1 — Additional file 1: Curriculum of the DEGRO-Academy, Version 2018. [file 13014_2021_1785_MOESM1_ESM.docx]

Curriculum der DEGRO-Akademie (2018)

1. Strahlenbiologie
   1. Grundlagen der molekularen und zellulären Strahlenwirkung: u.a. Zellüberleben, DNA- Reparatur, Zellzyklus, Modifikation der Strahlenantwort, LET, RBW, Signaltransduktion
   2. Grundlagen der Strahlenbiologie von Tumor- und Normalgewebe: u.a. 5 Rs der Strahlenbiologie, neue Erkenntnisse der Tumorbiologie, Hypoxie, Mikromilieu, Immunbiologie
   3. Frühe Normalgewebsreaktionen: (ZNS, Herz, Lunge, Niere, Leber, Pankreas, Haut, Knochen, Weichteile, Harnblase, Darm, Sinnesorgane)
   4. Fraktionierung, Hypofraktionierung, Stereotaxie
   5. Gesamtbehandlungszeit
   6. Volumeneffekt und NTCP
   7. Re-Bestrahlung
   8. Späte Normalgewebsfolgen: Organ-spezifisch (ZNS, Herz, Lunge, Niere, Leber, Pankreas, Haut, Knochen, Weichteile, Harnblase, Darm, Sinnesorgane)
   9. Strahlenwirkung auf Fortpflanzungsorgane und Strahlenrisiko allgemein
   10. Kombinierte Radiochemotherapie: u.a. Wechselwirkungen zwischen Radiotherapie und Chemotherapeutika
   11. Molekulares und biologisches Targeting: u.a. grundlegendes Konzept, Target-Identifizierung, Target-Validierung
   12. Individualisierung und Personalisierung der Strahlentherapie: u.a. Omics, Definition von prognostischen und prädiktiven Markern
   13. Molekulare, zelluläre und gewebsspezifische Effekte durch Partikeltherapie
2. Physik und Strahlenschutz
   1. Strahlenschutzrecht
   2. Grundlagen des Strahlenschutzes
   3. Strahlenschutzgrundsätze
      1. Rechtfertigung, Dosisbegrenzung, Vermeidung
   4. Organisation des betrieblichen Strahlenschutzes
   5. Natürliche und zivilisatorische Strahlenexposition
   6. Strahlenschutzbereiche, Grenzwerte für Patient, Personal und Umgebung, Personendosimetrie
   7. Stochastische und deterministische Strahlenwirkungen
   8. Fehler- und Risikomanagement
   9. Struktur der Materie
   10. Ionisierende Strahlung, Strahlenarten, Radioaktivität, Strahlerzeugung
   11. Radionuklide, Grundlagen der Nuklearmedizin
   12. Dosisbegriffe, Dosismessung
   13. Wechselwirkung von Strahlung und Materie, Dosisverteilungen
   14. Technische Evolution der Strahlentherapie / historische Betrahlungsgeräte
   15. Aufbau und Funktionsweise von med. Linearbeschleunigern und Kollimatoren
   16. Volumendefinition nach ICRU
   17. Dosisberechnungsalgorithmen
   18. Bestrahlungstechniken in der Teletherapie / 3D-Betrahlungsplanung
   19. Intensitätsmodulierte Techniken / VMAT/ Tomotherapie
   20. Planqualitätsanalyse: Isodosen, DVH, Konformität, Homogenität
   21. Biologische Bestrahlungsplanung
   22. Planverifikation: Gamma-Index-Analysen, DVH-Vergleich
   23. Simulation: klassisch, virtuell, am CT
   24. IGRT
   25. Gating und adaptive Strahlentherapie
   26. Brachytherapie: technische Voraussetzungen / Gerätetechnik, Volumendefinition, Bestrahlungsplanung, Plananalyse, Applikation
   27. Spezielle Bestrahlungstechniken: IORT, stereotaktische Strahlenbehandlung, Strahlenbehandlung der gesamten Haut
   28. Therapie mit Protonen, Neutronen, Schwerionen etc.
   29. Grundlagen der Bildgebung: Gerätetechnik, Rekonstruktion, Fusionierung, Registrierung, Segmentierung
3. Strahlentherapeutischer Techniken
   1. HDR- & Brachytherapie inkl. Seed-Implantation
   2. Bestrahlungsplanung in der Brachytherapie
   3. Spezifikation der Dosisverteilung im Zielvolumen bei der Brachytherapie
   4. 3D-Planung, virtuelle und CT-Simulation
   5. GTV, CTV, PTV und relevante UCRU- bzw. DIN-Empfehlungen
   6. Zielvolumendefinition und Konturierung, Bestimmung des Zielvolumens in der klinischen Praxis
   7. Spezifikation der Dosisverteilung im Zielvolumen bei externer RT
   8. Typische Bestrahlungstechniken
   9. Prinzipien, Techniken und Anwendungen der konformalen RT und IMRT/VMAT inkl. ICRU50, IRCU62 und ICRU83 Reports
   10. Besondere Techniken: Ganzkörperbestrahlung, Strahlenbehandlung der gesamten Haut, Ruthenium-Augenschalen
   11. IORT mittels Röntgenstrahlung bzw. Elektronen
   12. Stereotaktische Bestrahlungstechniken: kraniell und extrakraniell
   13. Strahlenbehandlung mit Protonen/Schwerionen, Prinzipien und klinische Anwendungen
   14. Modalitäten der Bildgebung, Verfahren, Technologie: z.B. Computertomographie, Ultraschall, MRT,PET
   15. Entwicklungen in der Bildgebung: z.B. PET-CT
   16. MR-bildgeführte Radiotherapie (MRgRT)
   17. Image-guided Radiotherapy (IGRT)
   18. Ultraschall-basierte IGRT
   19. IGRT mittels Oberflächen-Scanning
   20. Deep-Inspiration Breathhold-Techniken: z.B. beim Mammakarzinom
   21. Gating/Tracking
   22. Kombinierte Strahlentherapie und Hyperthermie
4. Klassifizierung von Akut- und Spätreaktionen, Supportivtherapie
   1. Radiogene Akut- und Spätreaktionen der in der klinischen Radioonkologie wesentlichen Organe / Organsysteme: Klinische Manifestierung sowie Grundlagen biologischer Mechanismen sowie deren Klassifizierung nach einer international gängigen Klassifikation (z.B. CTCAE für Akut und LENT-SOMA für Spätreaktionen
   2. Toleranzdosen wesentlicher Risikoorgane: z.B. nach den evidenzbasierten Vorgaben der QUANTEC-Tabellen für die normofraktionierte Bestrahlung sowie die Toleranzdosistabellen der AAPMTG (American Association of Physicists in Medicine Task Force) für die hypofraktionierte Bestrahlung
   3. Supportivtherapie bei radiogenen Akut- sowie Spätreaktionen wesentlicher relevanter Normalgewebe inkl. medikamentöser und physikalischer Begleitbehandlung (auch zur Protektion gesunder Gewebe) mit auch Evidenz-basierten komplementärmedizinischen Ansätzen
   4. Wesentliche Organsysteme, auf die sich die Punkte 5.1. bis 5.3. beziehen
      1. Haut / Schleimhaut / Unterhaut
      2. Muskeln / Knochen
      3. Knochenmark
      4. ZNS (Hirn / Retina / N. opticus / Chiasma / Innenohr / Hypophyse / Hirnstamm)
      5. Peripheres Nervensystem (Rückenmark / periphere Nerven (-plexus))
      6. Kopf-Hals-Bereich (Speicheldrüsen / Mandibula / Kiefergelenk / Zähne / Larynx / Schulddrüse)
      7. Herz / Gefäße
      8. Lunge
      9. Leber
      10. Gastrointestinaltrakt (Ösophagus / Magen / Dünndarm / Colon / Sigma / Rektum)
      11. Genito-Urethralsystem (Niere / ableitende Harnwege (Ureter, Urethra) / Harnblase / Hoden / Vulva/ Vagina / Uterus / Cervix / Ovarien)
   5. Grundlagen der Palliativmedizin, inkl.
      1. Schmerztherapie (u.a. medikamentös)
      2. Ernährungsmedizin / supportive Ernährungstherapie (enteral/parenteral)
      3. Psychoonkologie (psychogene Symptome / somatopsychische Reaktionen / psychosoziale Zusammenhänge)
      4. Supportivtherapie bei kombinierter Radiochemotherapie, Blutersatztherapie sowie sonstiger Infusionstherapie
   6. Rehabilitation / Nachsorge
5. Palliative Strahlentherapie
   1. Definition palliative Strahlentherapie
   2. Grundkenntnisse in der interdisziplinären Palliativtherapie
      1. Systemtherapie, Operative Therapie, Supportive Care
   3. Kenntnisse der grundsätzlichen Unterschiede zwischen kurativer und palliativer Strahlentherapie
   4. Kenntnisse über die wichtigsten Indikationen für eine palliative Strahlentherapie
      1. Knochenmetastasen
      2. Hirnmetastasen
      3. Fortgeschrittene Tumorerkrankungen (z.B. Bronchialkarzinom, Kopf-Hals-Tumoren)
      4. Hautmetastasen
      5. Lungenmetastasen
      6. Kompressionssyndrome
      7. Lebermetastasen, Lebermalignome
      8. Tumorblutung
      9. Rektumkarzinomrezidive
      10. Andere Tumorrezidive
      11. Splenomegalie
      12. Sonstige Situationen
   5. Kenntnis der unterschiedlichen Fraktionierungsschemata in der palliativen Strahlentherapie und der strahlenbiologischen Implikationen
      1. Hypofraktionierung, Akzelerierung, Einzeitbestrahlung
   6. Notfälle in der Strahlentherapie
      1. Obere Einflussstauung, Tumorblutungen, drohender Querschnitt, Hirndrucksymptomatik
      2. Grundkenntnisse der palliativen Strahlentherapie bei nicht-malignen Erkrankungen, z.B. Sialorrhoe bei amyotropher Lateralsklerose (ALS)
   7. Grundlagen der Palliativmedizin, inkl.
      1. Scherztherapie (u.a. medikamentöse)
      2. Ernährungsmedizin / supportive Ernährungstherapie (enteral sowie parenteral)
      3. Psychoonkologie (psychogene Symptome / somatopsychische Reaktionen / psychosoziale Zusammenhänge)
      4. Supportivtherapie, Blutersatztherapie sowie sonstiger Infusionstherapie
   8. Grundkenntnisse der Hospizarbeit
   9. Grundkenntnisse komplementärer und alternativer Medizin am Lebensende
6. Strahlentherapie bei gutartigen Erkrankungen
   1. Definition Strahlentherapie bei gutartigen Erkrankungen
   2. Unterscheidung maligne und nicht-maligne Erkrankungen
   3. Kenntnisse der grundsätzlichen Unterschiede zwischen der Strahlentherapie maligner und nicht-maligner Strahlentherapie
   4. Kenntnisse der Epidemiologie, Ätiologie und Pathogenese der wichtigsten gutartigen, nicht-malignen Erkrankungen
   5. Kenntnisse über die grundsätzlichen nicht-strahlentherapeutischen Behandlungsmöglichkeiten bei den wichtigsten gutartigen nicht-malignen Erkrankungen
   6. Kenntnisse über die wichtigsten Indikationen für eine Strahlentherapie bei nicht-malignen Erkrankungen
      1. Neurinom, Akustikusneurinom: Indikation und Behandlungskonzepte zur normofraktionierten und radiochirurgischen RT
      2. Meningeom, Hämangioperizytom: definitive und postoperative Behandlungskonzepte, u.a. in Abhängigkeit der histologischen WHO-Graduierung (Meningeome WHO Grade I, II und III)
      3. Hypophysentumore wie Adenome und Karzinome: Indikation und Behandlungskonzepte zur definitiven und postoperativen RT, Unterscheidung hormonproduzierend/nicht-hormonproduzierend
      4. Kraniopharyngeom: Indikation und Behandlungskonzepte zur definitiven und postoperativen RT
      5. Chordom: Indikation und Behandlungskonzepte zur definitiven und postoperativen RT
      6. Glomustumore /Chemodektome: Indikation und Behandlungskonzepte zur RT
      7. Arteriovenöse Malformation: Indikation und Behandlungskonzepte zur RT
   7. Kenntnisse der strahlenbiologischen Grundlagen der gutartigen, nicht-malignen Erkrankungen zur Verfügung stehenden indikationsabgestimmten, technischen strahlentherapeutischen Möglichkeiten: vom Orthovoltgerät über den modernen Linearbeschleuniger mit IMRT bis zur Stereotaxie
   8. Kenntnisse über die besonderen Erfordernisse des Strahlenschutzes bei gutartigen, nicht-malignen Erkrankungen
   9. Grundkenntnisse der Schmerzentstehung und der Schmerztherapie bei nicht-malignen Erkrankungen
   10. Grundkenntnisse des Vorgehens bei seltenen gutartigen Erkrankungen
   11. Herz-Radiochirurgie-Elektrophys.-basierte stereotakt. Strahlenth.arrythmogener kardialer Foci
7. Maligne onkologische organbezogene Entitäten inkl. Radiochemotherapie und targeted drugs
   1. Zentralnervensystem und Sinnesorgane - Maligne Tumore
      1. Niedriggradige Hirntumore WHO-Grad I und II wie beispielsweise Astrozytome und Oligodendrogliome: Indikation und Behandlungskonzepte zur definitiven und postoperativen RT, Indikation zur simultanen Systemtherapie
      2. Höhergradige Hirntumore WHO-Grad III und IV wie beispielsweise anaplastische Astrozytome und Glioblastome: Indikation und Behandlungskonzepte zur definitiven und postoperativen RT, Indikation zur simultanen Systemtherapie, u.a. in Abhängigkeit des molekularen Tumorprofils (MGMT-Hypermethylierung, IDH1/2- Mutation, 1p/19q-Ko-Deletion)
      3. Ependymale Tumore: Indikation und Behandlungskonzepte zur definitiven und postoperativen RT, Indikation zur Bestrahlung der Neuroachse
      4. Medulloblastom: Indikation und Behandlungskonzepte zur definitiven und postoperativen RT, Indikation zur Bestrahlung der Neuroachse
      5. Primäres Zerebrales Lymphom: Indikation und Behandlungskonzepte zur definitiven und postoperativen RT, Indikation zur simultanen Systemtherapie
      6. Sekundäre Hirntumore - Hirnmetastasen: Indikation und Behandlungskonzepte zur definitiven und postoperativen RT, Indikation Ganzhirnbestrahlung und Radiochirurgie
   2. Kopf-Hals
      1. Plattenepithelkarzinom der Mundhöhle, Oropharynx, des Larynx, des Hypopharynx: Indikation und Behandlungskonzepte zur definitiven und postoperativen RT, Indikation zur simultanen Systemtherapie inkl. Datenlage Radio-Antikörpertherapie, Konzepte zum Organerhalt, Hyperfraktionierung
      2. Plattenepithelkarzinome der Nebenhöhlen: Indikation und Behandlungskonzepte zur definitiven und postoperativen RT, simultane Systemtherapie
      3. Nasopharynxkarzinom: Indikation und Behandlungskonzepte zur definitiven und postoperativen RT, simultane Systemtherapie
      4. Adenokarzinome der Speicheldrüsen: Indikation und Behandlungskonzepte zur definitiven und postoperativen RT, Indikation zur simultanen Systemtherapie
      5. CUP-Syndrom: Indikation und Behandlungskonzepte zur definitiven und postoperativen, simultane Systemtherapie, Umfelddiagnostik / Staging
      6. Schilddrüsenkarzinom: Indikation und Behandlungskonzepte zur definitiven und postoperativen RT
   3. Mamma
      1. Invasives Adenokarzinom der Mamma (NST): Indikation und Behandlungskonzepte zur postoperativen RT nach brusterhaltender Operation und Mastektomie, Indikationsstellung zur RT der regionalen Lymphabflusswege, Normo-/Hypofraktionierung, Intra-operative RT (IORT), Interstitielle Brachytherapie
      2. Duktales Carcinoma-in-situ (DCIS) der Mamma: Indikation und Behandlungs-konzepte zur postoperativen RT
   4. Lunge
      1. Nicht-kleinzelliges Bronchialkarzinom: Indikation und Behandlungskonzepte zur prä- und postoperativen RT sowie definitiven RT in Abhängigkeit des TNM-Stadiums, simultane Systemtherapie, Stereotaktische (hypofraktionierte) RT
      2. Kleinzelliges Bronchialkarzinom: Indikation und Behandlungskonzepte zur prä- und postoperativen RT sowie definitiven RT in Abhängigkeit des TNM-Stadiums, simultane Systemtherapie, Stereotaktische (hypofraktionierte) RT, prophylaktische Ganzhirnbestrahlung
      3. Pleuramesotheliom: Indikation und Behandlungskonzepte zur prä- und postoperativen RT sowie definitiven RT in Abhängigkeit des TNM-Stadiums, simultane Systemtherapie
      4. Thymuskarzinom: Indikation und Behandlungskonzepte zur RT
      5. Thymom: Indikation und Behandlungskonzepte zur definitiven und postoperativen RT sowie definitiven RT in Abhängigkeit der Masaoka-Klassifikation
   5. Ösophaguskarzinom
      1. Plattenepithelkarzinom: Indikation und Behandlungskonzepte zur prä- und postoperativen RT sowie definitiven RT in Abhängigkeit des TNM-Stadiums und der Lokalisation (oberes, mittleres, unteres Drittel), simultane Systemtherapie
      2. Adenokarziom: Indikation und Behandlungskonzepte zur prä- und postoperativen RT sowie definitiven RT in Abhängigkeit des TNM-Stadiums, simultane Systemtherapie
   6. Adenokarzinom des Magens Indikation und Behandlungskonzepte zur prä- und postoperativen RT sowie definitiven RT in Abhängigkeit des TNM-Stadiums, simultane Systemtherapie
   7. Adenokarzinom des Pankreas Indikation und Behandlungskonzepte zur prä- und postoperativen RT sowie definitiven RT in Abhängigkeit der Operabilität und Vollständigkeit der Resektion, simultane Systemtherapie
   8. Hepatobiliäres System
      1. Cholangiozelluläres Karzinom: Indikation und Behandlungskonzepte zur prä- und postoperativen RT sowie definitiven RT in Abhängigkeit des TNM-Stadiums und der Lokalisation (intra-/extrahepatisch), simultane Systemtherapie, Stereotaktisch (hypofraktionierte) RT (intrahepatische Lokalisation)
      2. Hepatozelluläres Karzinom: Indikation und Behandlungskonzepte RT, Stereotaktisch (hypofraktionierte) RT
      3. Lebermetastasen: Stereotaktisch (hypofraktionierte) RT
   9. Adenokarzinom des Rektums: Indikation und Behandlungskonzepte zur prä- und postoperativen RT (sowie definitiven RT im individuellen Fall) in Abhängigkeit des TNM-Stadiums, der Lokalisation (oberes, mittleres, unteres Drittel), simultane Systemtherapie, kurzes und langes Konzept
   10. Plattenepithelkarzinom des Analkanals Indikation und Behandlungskonzepte zur definitiven und postoperativen RT, simultane Systemtherapie
   11. Niere und ableitende Harnorgane
       1. Nierenzellkarzinom: Indikation und Behandlungskonzepte zur postoperativen RT (sowie definitiven RT im individuellen Fall)
       2. Harnblasenkarzinom: Indikation und Behandlungskonzepte zur postoperativen RT (sowie definitiven RT im individuellen Fall), simultane Systemtherapie, Organerhalt
       3. Urothelkarzinom: Indikation und Behandlungskonzepte zur postoperativen RT (sowie definitiven RT im individuellen Fall), simultane Systemtherapie
       4. Peniskarzinom: Indikation und Behandlungskonzepte zur postoperativen RT sowie definitiven RT, simultane Systemtherapie, Organerhalt
   12. Prostatakarzinom: Indikation und Behandlungskonzepte zur postoperativen RT sowie definitiven RT, Indikation zur antihormonellen Therapie, Salvage-RT, Interstitielle Brachytherapie im Afterloading-Verfahren mit 192Iridium, Interstitielle Brachytherapie mit Permanentstrahlern
   13. Hodentumore
       1. Seminom: Indikation und Behandlungskonzepte zur postoperativen RT sowie definitiven RT abhängig vom TNM-Stadium
       2. Nicht-seminomatöse Keimzelltumore: Indikation und Behandlungskonzepte zur postoperativen RT sowie definitiven RT abhängig vom TNM-Stadium, Indikation zur RT zerebraler Metastasen
       3. Testikuläre intraepitheliale Neoplasie (TIN): Indikation und Behandlungskonzepte zur RT
   14. Gynäkologische Tumore des Beckens
       1. Plattenepithelkarzinom der Cervix uteri: Indikation und Behandlungskonzepte zur postoperativen RT sowie definitiven RT, simultane Systemtherapie, Interstitielle Brachytherapie im Afterloading-Verfahren mit 192Iridium als Boostkonzept
       2. Adenokarzinom des Corpus uteri (Endometriumkarzinom): Indikation und Behandlungskonzepte zur postoperativen RT (sowie definitiven RT im individuellen Fall), adjuvante RT als Brachytherapie im Afterloading-Verfahren mit 192Iridium
       3. Plattenepithelkarzinom der Vulva: Indikation und Behandlungskonzepte zur postoperativen RT sowie definitiven RT, simultane Systemtherapie, RT als Brachytherapie im Afterloading-Verfahren mit 192Iridium
       4. Vaginalkarzinom: Indikation und Behandlungskonzepte zur postoperativen RT sowie definitiven RT, simultane Systemtherapie, RT als Brachytherapie im Afterloading-Verfahren mit 192Iridium
       5. Ovarialkarzinom: Indikation und Behandlungskonzepte zur postoperativen RT sowie definitiven RT, simultane Systemtherapie, Ganz-Abdomen-Bestrahlung im Einzelfall
   15. Weichteilsarkome
       1. Extremitätensarkome: Indikation und Behandlungskonzepte zur prä- und postoperativen RT sowie definitiven RT, RT als Interstitielle Brachytherapie im Afterloading-Verfahren mit 192Iridium als Boostkonzept, Organerhalt
       2. Retroperitoneale Sarkome: Indikation und Behandlungskonzepte zur prä- und postoperativen RT
       3. Desmoide/aggressive Fibromatose: Indikation und Behandlungskonzepte zur postoperativen RT
   16. Lymphome:
       1. Morbus Hodgkin: Indikation und Behandlungskonzepte zur RT im Rahmen multimodaler Konzepte
       2. Non-Hodgkin-Lymphome: Indikation und Behandlungskonzepte zur RT im Rahmen multimodaler Konzepte
       3. Multiple Myelome und Plasmozytom: Indikation und Behandlungskonzepte zur RT im Rahmen multimodaler Konzepte oder als definitive Therapie
   17. Hauttumore
       1. Basalzellkarzinom und Plattenepithelkarzinom: Indikation und Behandlungskonzepte zur postoperative sowie definitiven RT, simultane Systemtherapie
       2. Malignes Melanom: Indikation und Behandlungskonzepte zur postoperative sowie definitiven RT, adjuvante RT der Lymphabflusswege
       3. Merkelzellkarzinom: Indikation und Behandlungskonzepte zur postoperative RT, adjuvante RT der Lymphabflusswege
   18. Tumore bei Kindern
       1. Osteosarkom, Ewing-Sarkom: Indikation und Behandlungskonzepte zur prä- und postoperative sowie definitiven RT im Rahmen multimodaler Konzepte, Ganzlungenbestrahlung
       2. Retinoblastom, Neuroblastom, Nephroblastom: Indikation und Behandlungskonzepte zur RT im Rahmen multimodaler Konzepte
       3. Leukämien: Indikation und Behandlungskonzepte zur Ganzkörperbestrahlung (GKB) im Rahmen multimodaler Konzepte, Dosierungskonzepte, Techniken zur GKB
       4. Knochenmetastasen: Indikation und Behandlungskonzepte zur RT, Einsatz eines adäquaten Fraktionierungsschemas, Vorgehen bei Rückenmarkskompression, Re-Bestrahlung
   19. Pterygium, Aderhaut-Hämangiom, endokrine Orbitopathie: Indikation und Behandlungskonzepte zur RT
   20. Entzündliche Veränderungen des Bewegungsapparates
       1. Bursitis, Tendinitis, Peritendinopathia humeroscapularis, Epicondylpathia hu-meri, Kalkaneodynie, Achillodynie, Osteoarthrosis deformans: Indikation und Behandlungskonzepte zur RT, Beachtung Strahlenschutz
   21. Erkrankungen von Bindegewebe und der Haut
       1. Induratio penis plastica, Morbus Dupuytren, Morbus Ledderhose, Keloi-de/hypertrophe Narben: Indikation und Behandlungskonzepte zur RT
   22. Heterotrophe Ossifikationen: Indikation und Behandlungskonzepte zur prä- und postoperativen RT
8. Bildgebung in der Strahlentherapie
   1. Grundsätzliche Kenntnisse und Fertigkeiten in der Bildgebung, die für die Bestrahlungsplanung notwendig sind
   2. Kenntnisse der technischen Grundlagen für die wichtigsten bildgebenden Verfahren (Röntgen, Ultraschall, CT, MRT, PET, Szintigraphie)
   3. Grundkenntnisse über den Einsatz von verschiedenen Kontrastmitteln
   4. Grundkenntnisse der Möglichkeiten der interventionellen Radiologie
   5. Kenntnisse der wichtigsten radiologischen und nuklearmedizinischen Befunde bei den wichtigsten Indikationen für eine Strahlentherapie
      1. Mammakarzinom
      2. Prostatakarzinom
      3. Bronchialkarzinom
      4. Rektumkarzinom
      5. Weitere gastrointestinale Tumoren
      6. Analkarzinom
      7. Kopf-Hals-Tumoren
      8. Hirntumoren
      9. Lymphome und Leukämien
      10. Weichteiltumoren
      11. Tumoren der Haut und Anhangsgebilde
      12. Gynäkologische Tumoren
      13. Urogenitale Tumoren
      14. Kindliche Tumoren
      15. Metastasen der verschiedenen Regionen
      16. Gutartige Erkrankungen
9. BVDST-relevante Komplexe, Abrechnung/DRG
   1. Organisation strahlentherapeutischer Einrichtungen (Klinik, MVZ, Praxis)
      1. Arbeitsrecht
      2. Personalbedarf und -planung
      3. Implementierung der digitalen Patientenakte
      4. Archivierung
   2. Qualitätsmanagement
      1. ISO 9001 und vergleichbare Instrumente
   3. Abrechnung mit besonderer Berücksichtigung moderner Therapieverfahren (IMRT, IGRT, etc.)
      1. EBM
      2. GOÄ
      3. DRG
   4. Rechtliche Grundlagen und deren konkrete Umsetzung
      1. Richtlinie Strahlenschutz
      2. Röntgenverordnung
   5. Ärztliche Stelle
      1. Kenntnisse und Anforderungen
      2. Auditvorbereitung
   6. Interdisziplinäre onkologische Versorgung
      1. Organzentren, Onkologische Zentren, Tumorzentren
      2. Aktuelle Konzepte (z.B. SAPV, ASV)
   7. Kooperation mit relevanten Institutionen im Gesundheitswesen
      1. Kassenärztliche Vereinigung
      2. Deutsche Krankenhausgesellschaft
      3. Private und gesetzliche Krankenkassen
   8. Öffentlichkeitsarbeit (Webpräsenz, soziale Medien, Printmedien)
      1. Von BVDST und DEGRO
      2. Der eigenen Institution
   9. Wissenschaftliches Arbeiten im nicht-universitären Umfeld
      1. Organisation und Durchführung von Studien
10. Sonstiges
    1. Historie der Radiologie und Strahlentherapie
       1. Entdeckung der Röntgenstrahlung
       2. Prinzipien der Teletherapie und ihre Entwicklung
       3. Strahlentherapie im Nationalsozialismus
    2. Prävention
    3. Epidemiologie und Statistik, Studienplanung
    4. Risikomanagement in der Radioonkologie
    5. Grundlagen der evidenzbasierten Medizin
    6. Ethische und legale Aspekte der Radioonkologie
    7. Volks- und betriebswirtschaftliche Aspekte der Radioonkologie
